# Association of modifiable metabolic risk factors and lifestyle with all-cause mortality in patients with hepatocellular carcinoma

**DOI:** 10.1038/s41598-024-65127-9

**Published:** 2024-07-04

**Authors:** Hwi Young Kim, Hye Ah Lee, Pompilia Radu, Jean-François Dufour

**Affiliations:** 1 Department of Internal Medicine, College of Medicine, Ewha Womans University, 1071, Anyangcheon-ro, Yangcheon-gu, Seoul, 07985 Republic of Korea; 2 Clinical Trial Center, Ewha Womans University Medical Center, Seoul, Republic of Korea; 3 University Clinic for Visceral Surgery and Medicine, University of Bern, Inselspital, Bern, Switzerland; 4 Centre for Digestive Diseases, Lausanne, Switzerland

**Keywords:** Hepatocellular carcinoma, Lifestyle, Comorbidity, Survival, Gastroenterology, Hepatology

## Abstract

We aimed to investigate the potential impact of metabolic risk factors and lifestyles on mortality in hepatocellular carcinoma (HCC) patients. From the Korean Central Cancer Registry database (2008–2016), 8,505 HCC patients were included in the analysis. Patients with 2 or more metabolic risk factors (n = 2384, 28.0%) showed significantly worse overall survival (OS, 29 months, 95% confidence interval [CI] 27–33) than patients with 0 (n = 2269 [26.7%]; 41 months, 95% CI 37–47), or 1 (n = 3852 [45.3%]; 42 months; 95% CI 38–46) metabolic risk factor. (*P* < 0.001) In the multivariable Cox analysis, patients with ≥ 2 metabolic risk factors had significantly elevated risk of overall mortality (adjusted hazards ratio (HR) = 1.14 [95% CI 1.06–1.23], *P* < 0.001) and HCC-specific mortality (sub-distribution HR = 1.09 [95% CI 1.00–1.09], *P* = 0.046), compared to those without. Alcohol and smoking were also independent risk factors for worse overall and HCC-specific mortality (all *P* < 0.05). Metabolic comorbidities were associated with greater risk of mortality in a dose-dependent manner in HCC patients, regardless of tumor stage and liver function. Alcohol intake and smoking significantly increased mortality by themselves and even further with the presence of metabolic risk.

## Introduction

Hepatocellular carcinoma (HCC) remains the sixth most common malignancy and the third most common cause of cancer-related mortality worldwide^1^. The main risk factors for HCC include chronic hepatitis C (HCV) or B virus (HBV) infection, alcohol-related liver disease, metabolic dysfunction-associated steatotic liver disease (MASLD), and type 2 diabetes^2^. Of those etiologies, MASLD has become the leading cause of chronic liver disease, with a global prevalence of approximately 30% in the general population^3^. Consequently, metabolic dysfunction-associated steatohepatitis (MASH) has been identified as the fastest rising cause of liver cancer incidence and death^4^.

Patients with MASLD or MASH may have variable phenotypic expression, including metabolic comorbidities such as obesity, metabolic syndrome, and diabetes^5^. These comorbidities increase the risk of HCC development^6,7^. In addition, metabolic comorbidities can modify the disease course of coexisting liver diseases, facilitating the development of HCC. For example, an independent association between diabetes and HCC risk was enhanced with comorbid metabolic conditions from a large U.S. population-based study^8^. New-onset diabetes and high body mass index (BMI) were associated with increased risk of HCC in patients with chronic HBV infection^9,10^. Moreover, lifestyle risk factors, such as alcohol intake, smoking, and physical inactivity, are also linked to HCC development^11^.

A recent study addressed the association between the metabolic risk burden and increased risk of HCC and all-cause mortality in patients with chronic hepatitis B^12^. However, effect of metabolic comorbidities on the survival of patients with HCC, in addition to their effect on HCC development, remains largely unknown. Given the recent advances in antiviral therapies against HCV and HBV, and the recent rise of MASLD as an important etiology of HCC, relevance of metabolic comorbidities in terms of survival needs to be evaluated from the perspective of disease prevention. In the present study, we aimed to investigate the potential impact of metabolic comorbidities on the overall survival (OS) of HCC patients in a nationwide cancer registry-based cohort.

## Results

### Baseline characteristics

Table 1 summarizes the baseline characteristics of the study population. Mean age at HCC diagnosis was 60.3 ± 11.2 years, and 6,781 patients were male (79.7%). Cirrhosis was present in 5668 patients (66.6%) at baseline. Most patients were classified as Child–Pugh class A (6501, 77.1%) or B (1618, 19.2%). Barcelona Clinic Liver Cancer (BCLC) stages were 0 or A in 3,403 patients (40.0%), B in 1,530 (18.0%), and C in 3154 (37.1%). Initial treatment modalities were surgical resection in 1,802 patients (21.2%), percutaneous ablation in 824 (9.7%), transarterial chemoembolization (TACE) in 3817 (44.9%), radiotherapy in 195 (2.3%), and systemic therapy in 540 (6.3%).

**Table 1:** Baseline characteristics of entire study cohort.

	Total (n = 8505)	No. of metabolic risk factors = 0 (n = 2269, 26.7%)	No. of metabolic risk factors = 1 (n = 3852, 45.3%)	No. of metabolic risk factors ≥ 2 (n = 2384, 28.0%)	*P*
Age	60.3 ± 11.2	56.3 ± 10.8	60.5 ± 10.9	63.9 ± 10.7	< 0.001
Sex (male)	6781 (79.7%)	1801 (79.4%)	3051 (79.2%)	1929 (80.9%)	0.234
ECOG					
0	5431 (78.1%)	1518 (79.8%)	2476 (78.7%)	1437 (75.4%)	< 0.001
1	1161 (16.7%)	305 (16.0%)	532 (16.9%)	324 (17.0%)	
2–4	363 (5.2%)	80 (4.2%)	138 (4.4%)	145 (7.6%)	
BMI	23.9 ± 3.3	23.2 ± 3.3	24.0 ± 3.3	24.5 ± 3.4	< 0.001
< 25.0	5569 (65.5%)	1679 (74.0%)	2508 (65.1%)	1382 (58.0%)	< 0.001
≥ 25.0	2936 (34.5%)	590 (26.0%)	1344 (34.9%)	1002 (42.0%)	
Smoking	3986 (46.9%)	1086 (47.9%)	1798 (46.8%)	1102 (46.3%)	0.502
Hypertension	5153 (60.6%)	0 (0%)	2796 (72.6%)	2357 (98.9%)	< 0.001
Diabetes	3158 (37.1%)	0 (0%)	924 (24.0%)	2234 (93.7%)	< 0.001
Hypercholesterolemia	395 (4.6%)	0 (0%)	132 (3.4%)	263 (11.0%)	< 0.001
Etiology					
HBV	5297 (62.3%)	1684 (74.3%)	2476 (64.4%)	1137 (47.8%)	< 0.001
HCV	857 (10.1%)	160 (7.1%)	424 (11.0%)	273 (11.5%)	
Alcohol	1092 (12.9%)	193 (8.5%)	450 (11.7%)	449 (18.9%)	
Others	1250 (14.7%)	231 (10.2%)	497 (12.9%)	522 (21.9%)	
Child–Pugh					
A	6501 (77.1%)	1716 (76.5%)	2992 (78.2%)	1793 (75.7%)	0.145
B	1618 (19.2%)	437 (19.5%)	692 (18.1%)	489 (20.7%)	
C	317 (3.8%)	89 (4.0%)	142 (3.7%)	86 (3.6%)	
BCLC					
0 A	3403 (40.0%)	956 (42.1%)	1579 (41.0%)	868 (36.4%)	< 0.001
B	1530 (18.0%)	345 (15.2%)	719 (18.7%)	466 (19.5%)	
C	3154 (37.1%)	856 (37.7%)	1377 (35.7%)	921 (38.6%)	
D	418 (4.9%)	112 (4.9%)	177 (4.6%)	129 (5.4%)	
AFP	35.5 (6.3–660)	48.3 (7.5–950)	32.9 (6.0–600)	30.4 (5.8–563.9)	< 0.001
Resection	1802 (21.2%)	507 (22.3%)	862 (22.4%)	433 (18.2%)	< 0.001
Local ablation	824 (9.7%)	225 (9.9%)	396 (10.3%)	203 (8.5%)	0.066
TACE	3817 (44.9%)	1010 (44.5%)	1696 (44.0%)	1111 (46.6%)	0.128
Radiation	195 (2.3%)	48 (2.1%)	91 (2.4%)	56 (2.3%)	0.804
Systemic therapy	540 (6.3%)	155 (6.8%)	245 (6.4%)	140 (5.9%)	0.407
Albumin	3.9 (3.4–4.2)	3.9 (3.4–4.3)	3.9 (3.4–4.3)	3.8 (3.3–4.2)	< 0.001
Bilirubin	0.9 (0.6–1.4)	1.0 (0.7–1.5)	0.9 (0.7–1.4)	0.9 (0.6–1.4)	< 0.001
INR	1.1 (1.0–1.2)	1.1 (1.0–1.2)	1.1 (1.0–1.2)	1.1 (1.0–1.2)	< 0.001
Creatinine	0.9 (0.7–1.0)	0.8 (0.7–1.0)	0.9 (0.7–1.0)	0.9 (0.8–1.1)	< 0.001
Sodium	139 (137–141)	139.6 (137–141)	139 (137–141)	138 (136–140)	< 0.001
Platelet	145 (100–201)	138 (92–193)	146 (101–202)	150 (107–210)	< 0.001

*ECOG* Eastern Cooperative Oncology Group, *BMI* body mass index, *IQR* interquartile range, *HBV* hepatitis B virus, *HCV* hepatitis C virus, *BCLC* Barcelona Clinic Liver Cancer, *AFP* alpha-fetoprotein, *TACE* transarterial chemoembolization, *INR* international normalized ratio.

**Figure 1:**
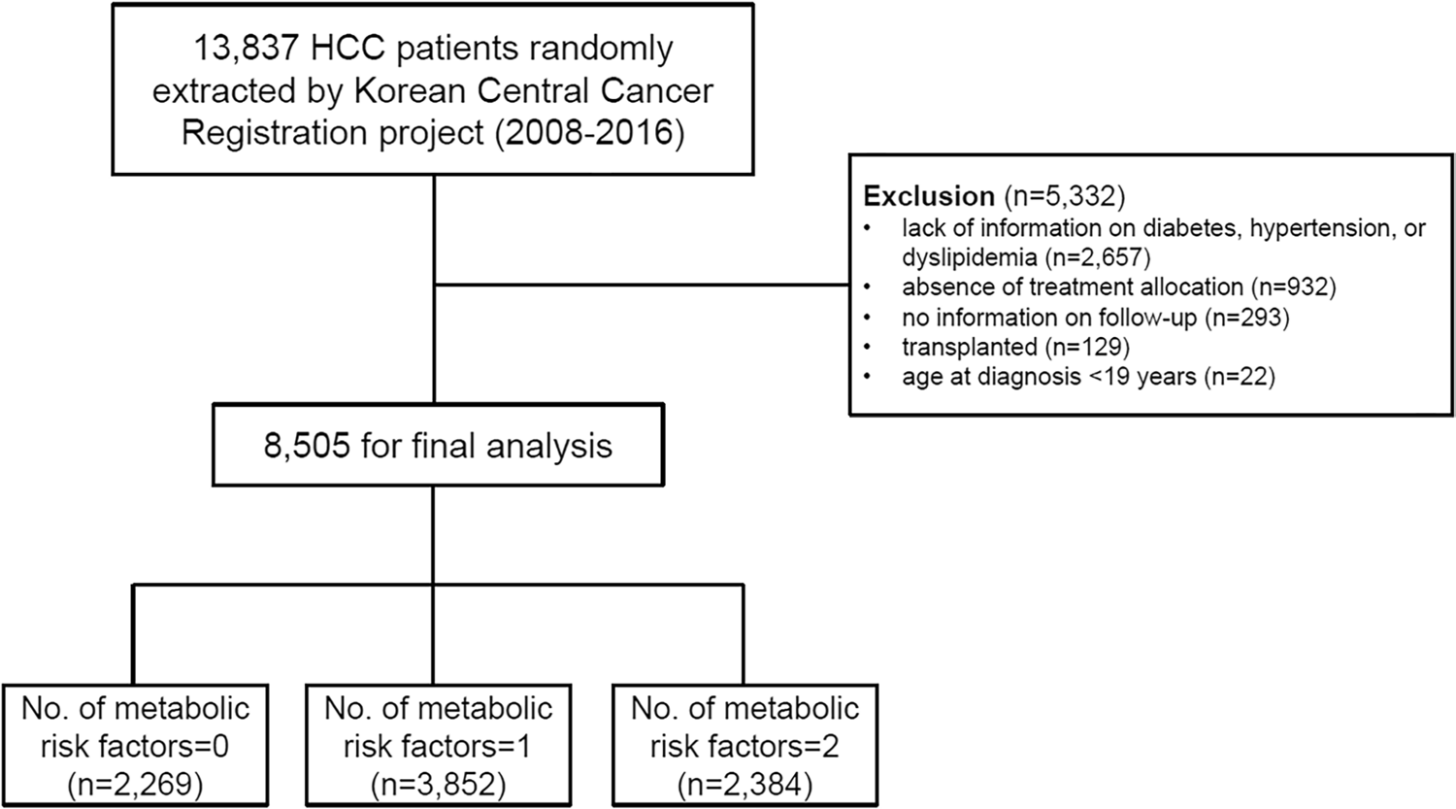
Flowchart of patient selection.

Metabolic comorbidities were identified at baseline as follows: hypertension in 5,153 patients (60.6%); diabetes in 3158 (37.1%); hypercholesterolemia, 395 (4.6%). History of smoking was reported in 3986 patients (46.9%). The numbers of patients with 0, 1, and ≥ 2 metabolic risk factors were 2269 (26.7%), 3852 (45.3%), and 2384 (28.0%), respectively. (Fig. 1) Patients with more metabolic risk factors were older, more likely to be alcoholic etiology, and more advanced tumor stage (all *P* < 0.05).

### Survival analysis: overall and HCC-specific mortality

**Figure 2:**
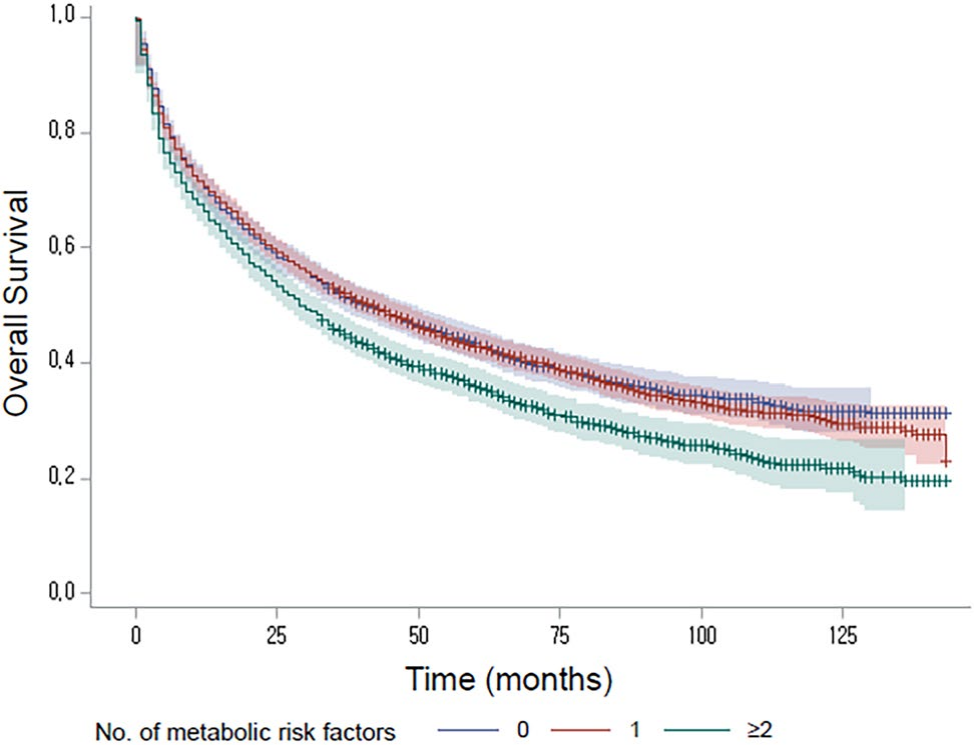
Kaplan–Meier curves for overall survival according to the number of metabolic risk factors (*P* < 0.001 by log-rank test).

The median follow-up duration of the entire study participants was 36 months (interquartile range [IQR], 8–69). During the follow-up period, 5493 patients died (64.6%), and the median overall survival (OS) was 37 months (95% CI 35–39). In patients without metabolic risk factor, the median OS was 41 months (95% confidence interval [CI] 37–47), which was not significantly different from the median OS of patients with 1 metabolic risk factor (median, 42 months; 95% CI 38–46; *P* = 0.608). However, patients with 2 or more metabolic risk factors (median, 29 months, 95% CI 27–33) showed significantly worse OS than patients with ≤ 1 metabolic risk factor. (*P* < 0.001 by log-rank test; Fig. 2) The burden of metabolic risk factor was positively associated with overall mortality (*P* for trend < 0.001).

In the multivariable Cox analysis (Table 2), patients with 2 or more metabolic risk factors had significantly elevated risk of overall mortality with an adjusted hazards ratio (HR) of 1.14 (95% CI 1.06–1.23; *P* < 0.001) compared to those without. When non-HCC deaths were considered as competing risks, the sub-distribution HR for HCC-specific mortality was 1.09 (95% CI 1.00–1.09; *P* = 0.046) for patients with 2 or more metabolic risk factors. Furthermore, alcohol intake and smoking were also identified as independent risk factors for worse overall and HCC-specific mortality (all *P* < 0.05).

**Table 2:** Uni- and multivariable Cox analysis for all-cause and HCC specific mortality.

	Unadjusted					
	HR	95% CI	*P*			
(A) Univariate analysis						
Age	1.01	(1.01–1.02)	< 0.001			
Sex						
Male	1.18	(1.11–1.27)	< 0.001			
Cirrhosis	1.43	(1.35–1.51)	< 0.001			
Child–Pugh						
A	Ref					
B	3.12	(2.93–3.32)	< 0.001			
C	4.53	(4.02–5.11)	< 0.001			
BCLC						
0, A	Ref					
B	2.42	(2.24–2.62)	< 0.001			
C	4.62	(4.32–4.94)	< 0.001			
D	7.97	(7.11–8.93)	< 0.001			
No. of metabolic risk factors						
0	Ref					
1	1.02	(0.95–1.09)	0.608			
≥ 2	1.25	(1.16–1.34)	< 0.001			
Trend	1.12	(1.08–1.16)	< 0.001			
BMI						
< 18.5	1.66	(1.46–1.88)	< 0.001			
18.5–24.9	Ref					
25.0–29.9	0.81	(0.76–0.85)	< 0.001			
≥ 30.0	0.86	(0.75–0.98)	0.023			
Alcohol	1.30	(1.23–1.37)	< 0.001			
Smoking	1.16	(1.10–1.23)	< 0.001			

	Multivariable-adjusted			Multivariable-adjusted*		
	HR	95% CI	*P*	SHR	95% CI	*P*
(B) Multivariate analysis						
Age	1.01	(1.01–1.02)	< 0.001	1.00	(1.00–1.01)	0.004
Sex						
Male	1.08	(1.00–1.16)	0.057	1.04	(0.95–1.13)	0.443
Cirrhosis	1.21	(1.13–1.29)	< 0.001	1.23	(1.15–1.32)	< 0.001
Child–Pugh						
A	Ref					
B	1.95	(1.82–2.10)	< 0.001	1.69	(1.55–1.83)	< 0.001
C	1.85	(1.45–2.36)	< 0.001	1.91	(1.35–2.69)	< 0.001
BCLC						
0, A	Ref					
B	2.27	(2.09–2.47)	< 0.001	2.47	(2.28–2.68)	< 0.001
C	3.87	(3.61–4.15)	< 0.001	3.97	(3.69–4.27)	< 0.001
D	4.46	(3.59–5.55)	< 0.001	3.37	(2.49–4.56)	< 0.001
No. of metabolic risk factors						
0	Ref					
1	1.02	(0.95–1.09)	0.657	1.02	(0.94–1.10)	0.625
≥ 2	1.14	(1.06–1.23)	< 0.001	1.09	(1.00–1.19)	0.046
Trend	1.07	(1.03–1.11)	< 0.001	1.05	(1.00–1.09)	0.045
BMI						
< 18.5	1.48	(1.30–1.68)	< 0.001	1.18	(0.99–1.40)	0.065
18.5–24.9	Ref			Ref		
25.0–29.9	0.83	(0.78–0.89)	< 0.001	0.88	(0.82–0.94)	< 0.001
≥ 30.0	0.84	(0.73–0.96)	0.012	0.90	(0.78–1.04)	0.155
Alcohol	1.08	(1.02–1.15)	0.009	1.08	(1.01–1.16)	0.027
Smoking	1.08	(1.02–1.15)	0.010	1.09	(1.01–1.17)	0.021

*Non-HCC deaths were considered as competing risks.

*BCLC* Barcelona clinic liver cancer, *BMI* body mass index, *HR* hazard ratio, *CI* confidence interval, *SHR* sub-distribution hazard ratio.

### Sensitivity analyses

The effect of metabolic risk factors on the OS was not significantly affected by initial treatment modalities (resection, TACE, local ablation, radiation, systemic therapy), after adjustment for age, sex, presence of cirrhosis, Child–Pugh class and BCLC stage (all *P* for heterogeneity > 0.05, Table 3). On the contrary, significant heterogeneity was observed with alcohol intake and smoking (Supplementary Table S1). Namely, risks of overall mortality increased gradually according to the metabolic risk burden in patients without alcohol intake or smoking, which was, however, not observed in patients with alcohol intake or smoking. Thus, we evaluated the effect of combined risks of alcohol intake/smoking and metabolic risk factors on the OS (Table 4). Compared with the reference subgroup (absence of metabolic risk factor and no alcohol intake/smoking), significant increase in the overall mortality risk was found in all except one subgroup with one metabolic risk and no alcohol intake/smoking. In the sensitivity analyses, significant effects of 2 or more metabolic risk factors on the OS were consistently observed in the subgroup of BCLC stage 0/A, Child–Pugh class A, and BCLC stage 0/A plus Child–Pugh class A (Supplementary Tables S2–S4). However, effects of metabolic risk factors on the HCC-specific mortality were not significant in these subgroups.

**Table 3:** Subgroup analysis according to the initial treatment modalities.

	No. of metabolic risk factors					*P* for heterogeneity	Trend	
	0	1		≥ 2				
		HR	95% CI	HR	95% CI		HR	95% CI
Resection								
No	Ref	1.03	(0.96–1.11)	1.15	(1.06–1.24)	0.110	1.07	(1.03–1.12)
Yes	Ref	0.86	(0.7–1.05)	0.90	(0.72–1.14)		0.95	(0.84–1.07)
TACE								
No	Ref	0.94	(0.86–1.04)	1.12	(1.01–1.25)	0.089	1.06	(1.01–1.12)
Yes	Ref	1.06	(0.97–1.17)	1.12	(1.01–1.24)		1.06	(1–1.11)
Local ablation								
No	Ref	1	(0.94–1.08)	1.11	(1.03–1.2)	0.497	1.06	(1.02–1.1)
Yes	Ref	0.93	(0.71–1.21)	1.21	(0.91–1.61)		1.1	(0.95–1.28)
Radiation								
No	Ref	1	(0.94–1.07)	1.12	(1.04–1.21)	0.609	1.06	(1.02–1.1)
Yes	Ref	0.85	(0.58–1.24)	1.11	(0.73–1.68)		1.06	(0.85–1.32)
Systemic therapy								
No	Ref	1	(0.93–1.07)	1.13	(1.05–1.22)	0.705	1.07	(1.03–1.11)
Yes	Ref	0.94	(0.76–1.17)	1.02	(0.8–1.29)		1.01	(0.89–1.14)

*Adjusted for age, sex, Child–Pugh class, BCLC stage, and underlying liver diseases.

*HR* hazard ratio, *CI* confidence interval, *TACE* transarterial chemoembolization.

**Table 4:** Combined effects of metabolic risk factors and alcohol intake/smoking on overall survival.

	No. of metabolic risk factors					
	0		1		≥ 2	
	HR	95% CI	HR	95% CI	HR	95% CI
Alcohol intake						
No	Ref		0.99	(0.91–1.08)	1.20	(1.09–1.32)
Yes	1.15	(1.03–1.29)	1.17	(1.06–1.29)	1.16	(1.05–1.29)
Smoking						
No	Ref		0.99	(0.9–1.09)	1.21	(1.09–1.34)
Yes	1.16	(1.04–1.29)	1.17	(1.06–1.29)	1.19	(1.07–1.32)

*HR* hazard ratio, *CI* confidence interval, *L-C* lifestyle-comorbidity.

## Discussion

In this nationwide cancer registry study, metabolic comorbidities were associated with greater risk of mortality in a dose-dependent manner. Patients with ≥ 2 metabolic risk factors (i.e., diabetes, hypertension and hypercholesterolemia) at the time of HCC diagnosis showed adjusted HR of 1.14 for all-cause mortality compared with those without metabolic risk factors, regardless of tumor stage and liver function. In addition, alcohol intake and smoking significantly increased mortality risk by themselves and with the presence of metabolic risk burden as well.

Baseline characteristics according to the number of metabolic risk factors showed distinctive features in the study participants. Patients with ≥ 2 metabolic risk factors were older, had higher BMI, more frequently alcoholic or other non-viral etiologies, and presented with more advanced stage, compared with those with less metabolic risk factors. In addition, they had slightly, but significantly worse hepatic function, and had higher platelet count. Patients with ≥ 2 metabolic risk factors were presumed to have MASLD as their underlying chronic liver disease. Patients with MASLD-HCC were older and more likely to present metabolic and cardiovascular comorbidities^13^. More advanced stages of these patients are probably related to their tendency toward later diagnosis due to lower disease awareness with resultant lower utilization of surveillance, as well as inadequate visualization on ultrasound even when they were subject to surveillance, compared to those with other etiologies^14,15^. Higher platelet count in these patients with ≥ 2 metabolic risk factors seems in line with studies suggesting that significant portion of HCC cases in the setting of MASLD develop in the absence of cirrhosis^16^.

Effect of metabolic comorbidities on the survival of HCC patients has been studied mostly in limited clinical settings so far. Previous studies have reported that the presence of diabetes predisposed worse prognosis of HCC patients. Increased mortality from liver cancer was associated with diabetes in a nationwide prospective study from China (relative risk, 1.54; 95% CI 1.28–1.86)^17^. Similarly, higher risks of HCC-specific mortality and all-cause mortality were associated with preexisting diabetes in meta-analyses^18–20^. However, diabetes, metabolic syndrome, obesity and MASLD are closely interrelated in their pathophysiology, as well as in hepatocarcinogenesis, given the common underlying mechanisms of insulin resistance-hyperinsulinemia, oxidative stress with DNA damage and chronic inflammation^21–24^. The aggregate effect of metabolic burden on the outcome of HCC has been mostly studied in selected patient groups with specific etiologies, particularly those with chronic hepatitis B. The aforementioned nationwide population-based study from Korea (n = 317,856) reported significant associations between increased metabolic risk burden and higher risks of HCC and all-cause mortality in patients with chronic hepatitis B, with multivariable-adjusted hazard ratios (HRs) of 1.13 (1.09–1.18) for 1 metabolic risk factor, 1.27 (1.22–1.33) for 2 metabolic risk factors, and 1.31 (1.23–1.39) for 3 metabolic risk factors, respectively^12^. Another study from Taiwan found similar association between metabolic risk factors and liver-related mortality (≥ 3 vs. 0 metabolic risk factors: adjusted HR, 2.72 [1.32–5.59]) in male HBV carriers (n = 1690)^25^. However, both studies were conducted in Asian patients with chronic hepatitis B, and the latter study assessed liver-related mortality in men instead of overall mortality of both sexes. Our results indicate a dose-dependent association of metabolic risk burden and all-cause mortality in Korean patients with HCC, after multivariable adjustment including etiology and tumor stage. Accordingly, metabolic risk burden needs to be assessed and managed in order to reduce the risk of HCC development as well as to improve the outcome.

The prognostic relevance of type 2 diabetes was suggested to depend on the stage of HCC. A meta-analysis reported differential impairment of overall survival after curative treatments in the presence of diabetes according to the tumor size (≤ 5 cm, HR = 1.63 [95% CI 1.25–2.1]; > 5 cm, HR = 0.67 [95% CI 0.39–1.15])^26^. Another study documented that diabetes was an independent prognostic factor only in patients within the Milan criteria and in patients with excellent performance status^27^. These data suggest that the prognostic relevance of diabetes in HCC patients may be more prominent in earlier stage. Considering the significance of tumor factors and treatment factors on the outcome of HCC patients, we conducted heterogeneity tests according to the initial treatment modalities. The effect of aggregate metabolic burden on the OS was not significantly affected by initial treatment modalities after adjustment (Table 3), suggesting that our results are not confined to selected patient subgroups treated with specific modalities. However, further validation is needed to verify consistent results as our findings in various clinical settings.

In addition, we conducted heterogeneity tests to evaluate effect modification by lifestyle factors, including alcohol intake and smoking, on the prognostic influence of the metabolic risk burden. Effect of metabolic risk burden on the OS, however, was not significant in the presence of either alcohol intake or smoking in the heterogeneity test as shown in Supplementary Table S1. Thus, combined effect of metabolic risk factors plus lifestyle on the OS was further investigated by regrouping patients into six mutually exclusive categories based on the combination of the presence of alcohol intake/smoking and the number of metabolic risk factors (Table 4). With the absence of both alcohol intake and metabolic risk factors as the reference, presence of alcohol intake or metabolic risk factor(s) demonstrated elevated risk of overall mortality, which was also consistent with smoking. Chiang et al. reported that HCC-specific mortality according to the presence of diabetes or smoking status in a nationwide prospective cohort from Taiwan was lowest in non-diabetic, never smokers, followed by non-diabetic, current smokers (HR = 2.49 [95% CI 1.71–3.64], and worst in diabetic, current smokers (HR = 4.73 [95% CI 2.44–9.17])^28^. A Swiss cohort study also reported that smoking was an independent risk factor on overall survival in patients with viral etiologies^29^. In addition, a strong effect modification by smoking was documented for the relationship between metabolic risk factors and HCC (*P* = 0.0044 for interaction) or liver-related death (*P* = 0.0015 for interaction) in the aforementioned cohort study of men with chronic hepatitis B from Taiwan, whereas no significant interaction was found for alcohol consumption^25^. However, alcoholic etiology showed either negative impact on survival due to delayed cancer detection and presence of advanced cirrhosis compared with HCV-HCC or similar outcomes as MASH-HCC^30,31^. A recent European hospital-based cohort study reported that alcohol use disorder contributed most to the liver disease burden in patients with type 2 diabetes, accounting for 57% of liver-related complications, including liver cancer and decompensated cirrhosis^32^. It seems premature to claim a conclusive theory regarding the complex interrelation between metabolic risk factors and modifiable lifestyle on the outcome of HCC patients. Nonetheless, our results showed that relevance of metabolic risk burden was consistent on the overall survival in the overall study participants as well as in the subgroup analyses, whereas the significance was not observed in HCC-specific mortality in the subgroup analysis. These lifestyle risk factors (i.e., alcohol intake and smoking) might have influenced synergistically with metabolic risk burdens on their survival, possibly causing deaths by acute lethal episodes such as cardiovascular events earlier than HCC-specific death. A Japanese study reported similar findings, with potential benefit of improving lifestyles on survival, particularly in patients with diabetes, hypertension and cancer^33^. Collectively, our findings as well as recent studies above underscore the importance of modifiable lifestyle adoption on top of metabolic risk management to improve outcome of HCC patients.

Our study has several limitations. First, the present study was conducted in Asian patients from a single country, which may not directly be extrapolated to other regions or racial groups. Second, some characteristics of metabolic syndrome was not captured from the Korea Central Cancer Registry (KCCR) registry due to lack of information, such as detailed lipid profile (triglycerides, low-or high-density lipoprotein cholesterol), or waist circumference. In addition, because data were measured at baseline, change in metabolic risk burden over time was not able to be assessed. Furthermore, several important points were not evaluated due to lack of data as follows: the impact of concurrent medications, such as aspirin, statin, or antidiabetic drugs, degree of diabetic control, characteristics of MASLD as etiology, and some demographic factors, such as education, occupation, and marital status^34,35^. Third, this study would be inherently subject to selection bias due to the retrospective study design. However, the KCCR has tried to minimize potential bias by using a random sampling audit method in this nationwide cancer registry. Fourth, there were no records on the subsequent treatment modalities, which prevented further analysis regarding the effect of secondary treatment on the outcome.

In conclusion, increased burden of metabolic comorbidities was associated with higher risk of all-cause mortality in a dose-dependent manner in patients with HCC, regardless of tumor stage and liver function. Moreover, alcohol intake and smoking significantly increased overall mortality risk with or without metabolic risk factors. Patients at risk of HCC need to be advised to control their metabolic disorders as well as to minimize/avoid alcohol intake and quit smoking.

## Materials and methods

### Database extraction

The KCCR is a nationwide cancer registry, which was initiated by the Ministry of Health and Welfare of South Korea in 1980. Patients with HCC were extracted from the KCCR registry using C22.0 of the International Classification of Disease 10th edition (ICD-10) coding system. The Korean Liver Cancer Association and National Cancer Center, Korea, have systemically organized the KCCR database on an annual basis by applying a random sample audit method. The KCCR registry covers a minimum of one of the 16 major administrative districts in Korea. A probability proportional extraction method was applied for the selection of hospitals in order to select hospitals with more patients with HCC by priority. Data were recruited for 83,231 patients between 2008 and 2016, by registering 11,547–12,194 patients from 47 to 54 hospitals across the country every year during the period (number varied by year.) Of these, records of 13,837 patients (13%) were randomly extracted, which included an additional 3% considering the presence of sampling errors. Clinical data for these 13,837 HCC patients were screened for eligibility for the present study.

Mortality data of the enrolled patients were provided by the Korean National Statistics Office. For survival analysis, dates of initial treatment were determined based on the KCCR records. Follow-up duration was estimated from the date of initial treatment till the date of death or December 31, 2019.

### Study population and clinical evaluation

A flow chart for the inclusion of the study participants is shown in Fig. 1. Of the 13,837 eligible patients from the KCCR database, 5332 patients were excluded as follows: lack of relevant information on diabetes, hypertension, and hypercholesterolemia (n = 3956); absence of treatment allocation (n = 932); no information on follow-up (n = 293); transplanted patients (n = 129); age < 19 years (n = 22). Finally, 8,505 patients were included in the analysis.

Based on the literature search^5–12^, data on the following variables were selected obtained from the KCCR database: age, sex, weight, height, history of smoking, comorbidities (hypertension, diabetes mellitus), etiology of HCC (HBV, HCV, alcohol, etc.), Child–Pugh class, tumor stage, alpha-fetoprotein (AFP), initial treatment modality (resection, local ablation, transarterial chemoembolization, radiation, systemic therapy), albumin, total bilirubin, prothrombin time (international normalized ratio), serum sodium (Na), and platelet count. Metabolic comorbidities were defined based on the diagnosis from the KCCR database and/or the following definitions: (i) diabetes: fasting blood glucose ≥ 126 mg/dL; (ii) hypercholesterolemia: total cholesterol level ≥ 240 mg/dL; (iii) hypertension: systolic/diastolic blood pressure ≥ 130/85 mm Hg^25,36^.

This study was conducted according to the ethical guidelines of the World Medical Association Declaration of Helsinki, and was approved by the Institutional Review Board of Ewha Womans University Hospital, Seoul, South Korea (approval No.: EUMC 2022-10-031). Informed consent was waived because the present study did not pose any harm to the study participants and no personally identifiable information was included.

### Statistical analysis

Baseline characteristics are expressed as frequencies and percentages for categorical variables and as means ± standard deviations for continuous variables. Analysis of variance and χ2 test were used to compare variables. OS was defined as the time from the date of the first HCC diagnosis to the time of death, the last follow-up evaluation, or the date of data censoring.

To investigate the impact of baseline metabolic risk profile on the all-cause mortality, we estimated adjusted HRs with 95% confidence intervals (CIs) using the multivariable Cox proportional hazard analysis. The adjusted covariables included age, sex, Child–Pugh class, BCLC stage, body mass index (BMI), alcohol intake, smoking, and the presence of chronic kidney disease (CKD). The assumptions of the Cox proportional hazards analysis were evaluated using the Schoenfeld residuals method. The Kaplan–Meier method with the log-rank test was used to compare OS between the subgroups according to the number of metabolic risk factors. Furthermore, a competing risk analysis was performed using the Fine and Gray model, considering deaths of non-HCC cause as a competing risk^37^. Sub-distribution hazard ratio (SHR) and 95% CIs were estimated. In addition, adjusted HRs for metabolic risk factors on the OS were calculated to evaluate heterogeneity according to (i) initial treatment modalities, and (ii) lifestyle (alcohol intake, smoking). For sensitivity analysis, we reiterated the analysis in patients with (i) BCLC stage 0/A, (ii) Child–Pugh class A, and iii) BCLC stage 0/A and Child–Pugh class A.

All tests were based on two-sided probability, and *P* < 0.05 was considered statistically significant. All analyses were performed using SAS 9.4 software (SAS Institute, Cary, NC, USA).

### Ethics declarations and Informed consent statement

This study was conducted according to the ethical guidelines of the World Medical Association Declaration of Helsinki, and was approved by the Institutional Review Board of Ewha Womans University Hospital, Seoul, South Korea (approval No.: EUMC 2022-10-031). Informed consent was waived because the present study did not pose any harm to the study participants and no personally identifiable information was included.

### Supplementary Information

Supplementary Tables.

## Data Availability

The datasets generated during and / or analysed during the current study are available from the corresponding author on reasonable request.

## References

[1] Sung H (2021). Global cancer statistics 2020: GLOBOCAN estimates of incidence and mortality worldwide for 36 cancers in 185 countries. CA Cancer J. Clin..

[2] Kulik L, El-Serag HB (2019). Epidemiology and management of hepatocellular carcinoma. Gastroenterology.

[3] Younossi ZM (2023). The global epidemiology of nonalcoholic fatty liver disease (NAFLD) and nonalcoholic steatohepatitis (NASH): A systematic review. Hepatology.

[4] Tan DJH (2023). Global burden of liver cancer in males and females: Changing etiological basis and the growing contribution of NASH. Hepatology.

[5] Younossi Z (2019). Global perspectives on nonalcoholic fatty liver disease and nonalcoholic steatohepatitis. Hepatology.

[6] Inoue M (2006). Diabetes mellitus and the risk of cancer: Results from a large-scale population-based cohort study in Japan. Arch. Intern. Med..

[7] Larsson SC, Wolk A (2007). Overweight, obesity and risk of liver cancer: A meta-analysis of cohort studies. Br. J. Cancer.

[8] Simon TG (2018). Diabetes, metabolic comorbidities, and risk of hepatocellular carcinoma: Results from two prospective cohort studies. Hepatology.

[9] Fu SC (2015). Increased risk of hepatocellular carcinoma in chronic hepatitis B patients with new onset diabetes: A nationwide cohort study. Aliment Pharmacol. Ther..

[10] Kim K, Choi S, Park SM (2018). Association of high body mass index and hepatocellular carcinoma in patients with chronic hepatitis B virus infection: A Korean population-based cohort study. JAMA Oncol..

[11] Saran U, Humar B, Kolly P, Dufour JF (2016). Hepatocellular carcinoma and lifestyles. J. Hepatol..

[12] Lee YB (2021). Association of metabolic risk factors with risks of cancer and all-cause mortality in patients with chronic hepatitis B. Hepatology.

[13] Huang DQ, El-Serag HB, Loomba R (2021). Global epidemiology of NAFLD-related HCC: trends, predictions, risk factors and prevention. Nat. Rev. Gastroenterol. Hepatol..

[14] Chong N (2022). Association between ultrasound quality and test performance for HCC surveillance in patients with cirrhosis: A retrospective cohort study. Aliment Pharmacol. Ther..

[15] Schoenberger H (2022). Dynamic changes in ultrasound quality for hepatocellular carcinoma screening in patients with cirrhosis. Clin. Gastroenterol. Hepatol..

[16] Orci LA (2022). Incidence of hepatocellular carcinoma in patients with nonalcoholic fatty liver disease: A systematic review, meta-analysis, and meta-regression. Clin. Gastroenterol. Hepatol..

[17] Bragg F (2017). Association between diabetes and cause-specific mortality in rural and urban areas of China. JAMA.

[18] Yang WS (2011). The role of pre-existing diabetes mellitus on hepatocellular carcinoma occurrence and prognosis: A meta-analysis of prospective cohort studies. PLoS One.

[19] Wang C (2012). Increased risk of hepatocellular carcinoma in patients with diabetes mellitus: A systematic review and meta-analysis of cohort studies. Int. J. Cancer.

[20] Wang P, Kang D, Cao W, Wang Y, Liu Z (2012). Diabetes mellitus and risk of hepatocellular carcinoma: A systematic review and meta-analysis. Diabetes Metab. Res. Rev..

[21] Park EJ (2010). Dietary and genetic obesity promote liver inflammation and tumorigenesis by enhancing IL-6 and TNF expression. Cell.

[22] Kudo Y (2011). Altered composition of fatty acids exacerbates hepatotumorigenesis during activation of the phosphatidylinositol 3-kinase pathway. J. Hepatol..

[23] Pollak M (2012). The insulin and insulin-like growth factor receptor family in neoplasia: An update. Nat. Rev. Cancer.

[24] Nakatsuka T (2017). Impact of histone demethylase KDM3A-dependent AP-1 transactivity on hepatotumorigenesis induced by PI3K activation. Oncogene.

[25] Yu MW (2017). Influence of metabolic risk factors on risk of hepatocellular carcinoma and liver-related death in men with chronic Hepatitis b: A large cohort study. Gastroenterology.

[26] Wang WM (2011). Prognostic role of diabetes mellitus in hepatocellular carcinoma patients after curative treatments: a meta-analysis. Hepatobiliary Pancreat. Dis. Int..

[27] Ho SY (2020). Differential survival impact of diabetes mellitus on hepatocellular carcinoma: Role of staging determinants. Dig. Dis. Sci..

[28] Chiang CH (2016). The relationship of diabetes and smoking status to hepatocellular carcinoma mortality. Medicine (Baltimore).

[29] Kolly P, Knopfli M, Dufour JF (2017). Effect of smoking on survival of patients with hepatocellular carcinoma. Liver Int..

[30] Bucci L (2016). Comparison between alcohol- and hepatitis C virus-related hepatocellular carcinoma: Clinical presentation, treatment and outcome. Aliment. Pharmacol. Ther..

[31] Kumar R, Goh BG, Kam JW, Chang PE, Tan CK (2020). Comparisons between non-alcoholic steatohepatitis and alcohol-related hepatocellular carcinoma. Clin. Mol. Hepatol..

[32] Mallet V (2022). Burden of liver disease progression in hospitalized patients with type 2 diabetes mellitus. J. Hepatol..

[33] Sakaniwa R (2022). Impact of modifiable healthy lifestyle adoption on lifetime gain from middle to older age. Age Ageing.

[34] Lee TY (2023). Daily aspirin associated with a reduced risk of hepatocellular carcinoma in patients with non-alcoholic fatty liver disease: A population-based cohort study. EClinicalMedicine.

[35] Jeon D (2023). Association between statin use and the prognosis of hepatocellular carcinoma after resection: A nationwide cohort study. EClinicalMedicine.

[36] Kim BH (2024). Advancing Korean nationwide registry for hepatocellular carcinoma: A systematic sampling approach utilizing the Korea Central Cancer Registry database. J. Liver Cancer.

[37] Fine JP, Gray RJ (1999). A proportional hazards model for the subdistribution of a competing risk. J. Am. Stat. Assoc..

