# Supplementary material for: Association of modifiable metabolic risk factors and lifestyle with all-cause mortality in patients with hepatocellular carcinoma
Source: Sci Rep. 2024 Jul 4;14:15405. doi: 10.1038/s41598-024-65127-9 (PMC11224327; doi:10.1038/s41598-024-65127-9)
Supplement: Supplementary file 1 — Supplementary Tables. [file 41598_2024_65127_MOESM1_ESM.docx]

**Supplementary Table 1.** Subgroup analysis according to alcohol intake and smoking

|  |  | No. of metabolic risk factors | | | | |  |  |  |
| --- | --- | --- | --- | --- | --- | --- | --- | --- | --- |
|  |  | 0 | 1 |  | ≥2 |  |  | Trend |  |
|  |  | HR | HR | 95% CI | HR | 95% CI | *P* for heterogeneity | HR | 95% CI |
| Alcohol intake | No | ref | 0.99 | (0.91–1.08) | 1.2 | (1.09–1.32) | 0.007 | 1.10 | (1.05–1.16) |
|  | Yes | ref | 1.01 | (0.91–1.13) | 1.01 | (0.90–1.13) |  | 1.00 | (0.95–1.06) |
| Smoking | No | ref | 0.99 | (0.9–1.09) | 1.21 | (1.09–1.34) | 0.015 | 1.11 | (1.05–1.17) |
|  | Yes | ref | 1.01 | (0.92–1.1) | 1.03 | (0.92–1.14) |  | 1.01 | (0.96–1.07) |

Abbreviations: HR, hazard ratio; CI, confidence interval; L-C, lifestyle-comorbidity.

**Supplementary Table 2.** Sensitivity analysis in patients with BCLC stage 0/A

|  |  | Multivariable-adjusted | | | Multivariable-adjusted | | | Multivariable-adjusted* | | |
| --- | --- | --- | --- | --- | --- | --- | --- | --- | --- | --- |
|  |  | HR | 95%CI | *P* | HR | 95%CI | *P* | HR | 95%CI | *P* |
| No. of metabolic risk factors | 0 | ref |  |  | ref |  |  | ref |  |  |
|  | 1 | 0.96 | (0.84–1.1) | 0.579 | 0.98 | (0.85–1.11) | 0.706 | 0.97 | (0.85–1.11) | 0.655 |
|  | ≥2 | 1.21 | (1.04–1.4) | 0.011 | 1.23 | (1.06–1.43) | 0.006 | 1.16 | (1.00–1.35) | 0.053 |
|  | Trend | 1.11 | (1.03–1.19) | 0.009 | 1.12 | (1.03–1.20) | 0.004 | 1.08 | (1.00–1.17) | 0.045 |
| Sex | Male | 1.12 | (0.99–1.27) | 0.065 | 1.12 | (0.99–1.27) | 0.077 | 1.01 | (0.88–1.16) | 0.942 |
| Age |  | 1.04 | (1.04–1.05) | <0.001 | 1.04 | (1.04–1.05) | <0.001 | 1.04 | (1.04–1.05) | <0.001 |
| LC |  | 1.52 | (1.34–1.73) | <0.001 | 1.52 | (1.34–1.73) | <0.001 | 1.6 | (1.41–1.82) | <0.001 |
| Child–Pugh | A | ref |  |  | ref |  |  | ref |  |  |
|  | B | 2.75 | (2.34–3.22) | <0.001 | 2.79 | (2.37–3.27) | <0.001 | 2.58 | (2.19–3.04) | <0.001 |
| BMI | <18.5 |  |  |  | 2.22 | (1.68–2.93) | <0.001 | 2.24 | (1.69–2.96) | <0.001 |
|  | 18.5–24.9 |  |  |  | ref |  |  | ref |  |  |
|  | 25.0–29.9 |  |  |  | 0.99 | (0.88–1.11) | 0.799 | 0.98 | (0.87–1.1) | 0.741 |
|  | ≥30.0 |  |  |  | 1.17 | (0.91–1.52) | 0.223 | 1.13 | (0.88–1.47) | 0.344 |
| Alcohol intake |  |  |  |  |  |  |  | 1.23 | (1.08–1.39) | 0.001 |
| Smoking |  |  |  |  |  |  |  | 1.09 | (0.96–1.23) | 0.205 |

*Non-HCC deaths were considered as competing risks.

Abbreviations: BCLC, Barcelona Clinic Liver Cancer; BMI, body mass index; HR, hazard ratio; CI, confidence interval; SHR, sub-distribution hazard ratio.

**Supplementary Table 3.** Sensitivity analysis in patients with Child-Pugh class A

|  |  | Multivariable-adjusted | | | Multivariable-adjusted | | | Multivariable-adjusted* | | |
| --- | --- | --- | --- | --- | --- | --- | --- | --- | --- | --- |
|  |  | HR | 95%CI | *P* | HR | 95%CI | *P* | HR | 95%CI | *P* |
| No. of metabolic risk factors | 0 | ref |  |  | ref |  |  | ref |  |  |
|  | 1 | 0.95 | (0.88–1.04) | 0.252 | 0.98 | (0.9–1.06) | 0.588 | 0.97 | (0.9–1.06) | 0.521 |
|  | ≥2 | 1.07 | (0.98–1.17) | 0.134 | 1.12 | (1.02–1.23) | 0.015 | 1.08 | (0.98–1.19) | 0.103 |
|  | Trend | 1.04 | (0.99–1.09) | 0.099 | 1.06 | (1.08–1.28) | 0.010 | 1.04 | (0.99–1.09) | 0.086 |
| Sex | Male | 1.19 | (1.09–1.29) | <0.001 | 1.17 | (1.08–1.27) | <0.001 | 1.08 | (0.98–1.18) | 0.125 |
| Age |  | 1.02 | (1.01–1.02) | <0.001 | 1.02 | (1.01–1.02) | <0.001 | 1.01 | (1.01–1.02) | <0.001 |
| LC |  | 1.18 | (1.11–1.27) | <0.001 | 1.19 | (1.11–1.27) | <0.001 | 1.21 | (1.13–1.29) | <0.001 |
| BCLC | 0, A | ref |  |  | ref |  |  | ref |  |  |
|  | B | 2.43 | (2.22–2.66) | <0.001 | 2.41 | (2.2–2.64) | <0.001 | 2.39 | (2.18–2.61) | <0.001 |
|  | C | 4.39 | (4.07–4.74) | <0.001 | 4.35 | (4.03–4.7) | <0.001 | 4.26 | (3.94–4.6) | <0.001 |
|  | D | 6.76 | (4.75–9.62) | <0.001 | 6.33 | (4.44–9.01) | <0.001 | 6.23 | (4.37–8.87) | <0.001 |
| BMI | <18.5 |  |  |  | 1.41 | (1.2–1.67) | <0.001 | 1.45 | (1.22–1.71) | <0.001 |
|  | 18.5–24.9 |  |  |  | ref |  |  | ref |  |  |
|  | 25.0–29.9 |  |  |  | 0.85 | (0.79–0.91) | <0.001 | 0.85 | (0.78–0.91) | <0.001 |
|  | ≥30.0 |  |  |  | 0.86 | (0.73–1.02) | 0.079 | 0.86 | (0.73–1.02) | 0.078 |
| Alcohol intake |  |  |  |  |  |  |  | 1.17 | (1.09–1.26) | <0.001 |
| Smoking |  |  |  |  |  |  |  | 1.05 | (0.97–1.13) | 0.234 |

*Non-HCC deaths were considered as competing risks.

Abbreviations: BCLC, Barcelona Clinic Liver Cancer; BMI, body mass index; HR, hazard ratio; CI, confidence interval; SHR, sub-distribution hazard ratio.

**Supplementary Table 4.** Sensitivity analysis in patients with BCLC stage 0/A and Child-Pugh class A

|  |  | Multivariable-adjusted | | | Multivariable-adjusted | | | Multivariable-adjusted* | | |
| --- | --- | --- | --- | --- | --- | --- | --- | --- | --- | --- |
|  |  | HR | 95%CI | *P* | HR | 95%CI | *P* | HR | 95%CI | *P* |
| No. of metabolic risk factors | 0 | ref |  |  | ref |  |  | ref |  |  |
|  | 1 | 0.89 | (0.78–1.03) | 0.124 | 0.9 | (0.78–1.04) | 0.167 | 0.90 | (0.78–1.04) | 0.156 |
|  | ≥2 | 1.17 | (1–1.36) | 0.054 | 1.19 | (1.01–1.39) | 0.035 | 1.13 | (0.96–1.33) | 0.141 |
|  | Trend | 1.09 | (1.00–1.18) | 0.039 | 1.1 | (1.01–1.19) | 0.025 | 1.07 | (0.98–1.16) | 0.112 |
| Sex | Male | 1.15 | (1.01–1.32) | 0.037 | 1.15 | (1.01–1.32) | 0.039 | 1.05 | (0.90–1.22) | 0.556 |
| Age |  | 1.05 | (1.04–1.05) | <0.001 | 1.05 | (1.04–1.05) | <0.001 | 1.04 | (1.04–1.05) | <0.001 |
| LC |  | 1.53 | (1.35–1.74) | <0.001 | 1.53 | (1.35–1.73) | <0.001 | 1.61 | (1.41–1.83) | <0.001 |
| BMI | <18.5 |  |  |  | 2.12 | (1.57–2.84) | <0.001 | 2.13 | (1.59–2.87) | <0.001 |
|  | 18.5–24.9 |  |  |  | ref |  |  | ref |  |  |
|  | 25.0–29.9 |  |  |  | 0.99 | (0.87–1.12) | 0.850 | 0.99 | (0.87–1.12) | 0.808 |
|  | ≥30.0 |  |  |  | 1.21 | (0.93–1.59) | 0.158 | 1.16 | (0.89–1.52) | 0.283 |
| Alcohol intake |  |  |  |  |  |  |  | 1.23 | (1.07–1.41) | 0.002 |
| Smoking |  |  |  |  |  |  |  | 1.04 | (0.91–1.19) | 0.547 |

*Non-HCC deaths were considered as competing risks.

Abbreviations: BCLC, Barcelona Clinic Liver Cancer; BMI, body mass index; HR, hazard ratio; CI, confidence interval; SHR, sub-distribution hazard ratio.
